# Complete genome sequencing and antibiotics biosynthesis pathways analysis of *Streptomyces lydicus* 103

**DOI:** 10.1038/srep44786

**Published:** 2017-03-20

**Authors:** Nan Jia, Ming-Zhu Ding, Hao Luo, Feng Gao, Ying-Jin Yuan

**Affiliations:** 1 Key Laboratory of Systems Bioengineering (Ministry of Education), School of Chemical Engineering and Technology, Tianjin University, Tianjin, 300072, P. R. China; 2 SynBio Research Platform, Collaborative Innovation Centre of Chemical Science and Engineering (Tianjin), School of Chemical Engineering and Technology, Tianjin University, Tianjin, 300072, P. R. China; 3 Department of Physics, Tianjin University, Tianjin, 300072, P. R. China

## Abstract

More and more new natural products have been found in *Streptomyces* species, which become the significant resource for antibiotics production. Among them, *Streptomyces lydicus* has been known as its ability of streptolydigin biosynthesis. Herein, we present the genome analysis of *S. lydicus* based on the complete genome sequencing. The circular chromosome of *S. lydicus* 103 comprises 8,201,357 base pairs with average GC content 72.22%. With the aid of KEGG analysis, we found that *S. lydicus* 103 can transfer propanoate to succinate, glutamine or glutamate to 2-oxoglutarate, CO_2_ and L-glutamate to ammonia, which are conducive to the the supply of amino acids. *S. lydicus* 103 encodes acyl-CoA thioesterase II that takes part in biosynthesis of unsaturated fatty acids, and harbors the complete biosynthesis pathways of lysine, valine, leucine, phenylalanine, tyrosine and isoleucine. Furthermore, a total of 27 putative gene clusters have been predicted to be involved in secondary metabolism, including biosynthesis of streptolydigin, erythromycin, mannopeptimycin, ectoine and desferrioxamine B. Comparative genome analysis of *S. lydicus* 103 will help us deeply understand its metabolic pathways, which is essential for enhancing the antibiotic production through metabolic engineering.

*Streptomyces* species are high-GC Gram-positive bacteria found predominantly in soil^1^. Through a complex process of morphological and physiological differentiation, *Streptomyces* species could produce many specialized metabolites used for agricultural antibiotics^2^. Some fungi can degrade difficult decomposition by lipase and cellulase, which play an important role in soil ecology^3^. Besides, the resistance genes of insecticide and herbicide in *Streptomyces* are widely used in transgenic plants^4^. These secondary metabolites are not essential for bacterial growth but have important roles in microbe-microbe communication^5^. As a root-colonizing actinomycete, *Streptomyces lydicus* can produce antibiotics or siderophore for suppressing fungal growth^6^. The elucidation of the related antimicrobial mechanism will facilitate the finding of novel antibiotics.

With the development of genome sequencing technology, more and more complete genomes of *Streptomyces* species have been announced. *S. lydicus* could produce streptolydigin which acts on catalytic function of RNA polymerase and inhibits RNA synthesis^7^. Our previous studies have identified its biosynthesis pathways of fatty acids^8^, type II thioesterase^9^ and nitrogen metabolism^10^ which are responsible for streptolydigin biosynthesis. Besides, proteomics and metabolomics approaches have been demonstrated in our previous studies on the responses of *S. lydicus* to pitching ratios during streptolydigin production^11^,^12^. However, only one complete genome sequence of *S. lydicus*, i.e., *S. lydicus* A02 (accession number CP007699.1), was available in GenBank. Therefore, we have carried out the complete genome sequencing of *S. lydicus* 103 and constructed its metabolic pathways of antibiotic biosynthesis, including primary metabolism and secondary metabolism. Previous work has shown that heterologously expression of *chit42* gene from *Trichoderma harzianum* P1 in *S. lydicus* A01 could enhance the chitinase activity and natamycin production^13^. Further functional characterization of the gene cluster will advance our understanding of the related pathways of antibiotic biosynthesis, and provide insight into the further analysis of the metabolism and gene targets for strain improvement.

## Results

### Genomic characteristics of *S. lydicus* 103

**Figure 1:**
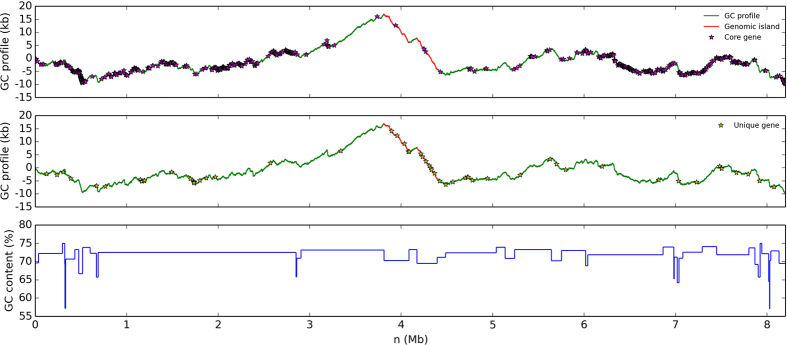
The GC profile of the genome of *S. lydicus* 103. With the aid of the distribution of core genes and unique genes, two large genomic islands (red lines) have been detected (GI-I: from 3812417 to 4085811 bp and GI-II: from 4171990 to 4395111 bp) in *S. lydicus* 103 based on GC-Profile. And the purple and yellow stars present core genes and unique genes of *S. lydicus* 103, respectively.

**Figure 2:**
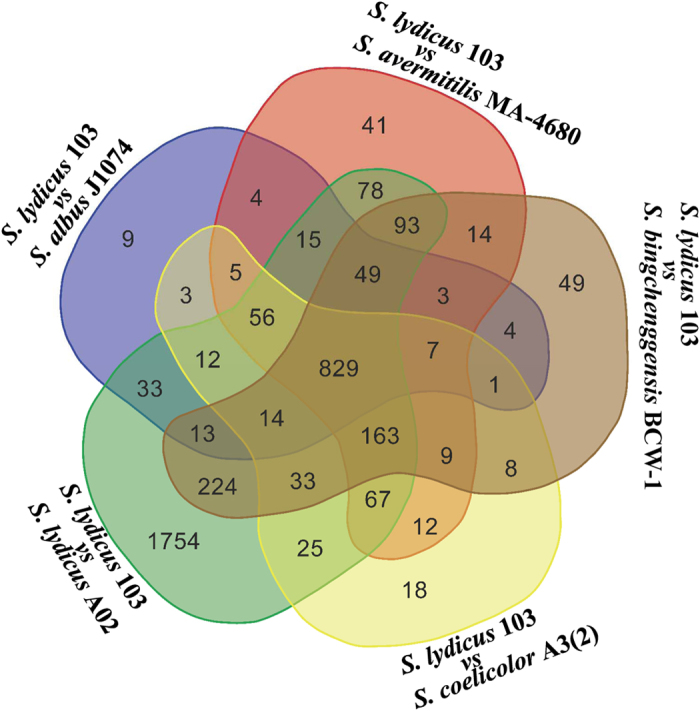
Venn diagram of the number of homologous genes between *S. lydicus* 103 and *S. albus* J1074, *S. avermitilis* MA-4680, *S. lydicus* A02, *S. coelicolor* A3(2) and *S. bingchenggensis* BCW-1, respectively. BioVenn, a web application for the comparison and visualization of biological lists, has been used for Venn diagrams drawing.

*S. lydicus* 103 has a chromosome of 8.20 Mb with 72.22% GC content, which contains 6,872 annotated protein-coding genes (Fig. S1 and Table S1). Mostly, the chromosomes of *Streptomyces* species are linear^14^. However, the chromosome of *S. lydicus* 103 in this study is circular, which may lead to more genetic stability. Phylogenetic analysis of *S. lydicus* 103 with other *Streptomyces* species has been carried out using CVTree (Fig. S2). BLASTP searches have been performed based on the whole amino acid sequences of *S. lydicus* 103 against those of other *Streptomyces* genomes listed in Fig. S2 with E-values less than 10^−5^. The protein-coding genes with the percent of identity and coverage larger than 80% in all *Streptomyces* genomes listed in Fig. S2 are defined as core genes (Figs 1 and 2). The protein-coding genes that have no hits within the other *Streptomyces* genomes listed in Fig. S2 are defined as unique genes. Based on the BLASTP results, 641 core genes and 59 unique genes in *S. lydicus* 103 are predicted by this definition. With the aid of the distribution of core genes and unique genes, two large genomic islands have been detected (GI-I: from 3812417 to 4085811 bp and GI-II: from 4171990 to 4395111 bp) in *S. lydicus* 103 based on GC-Profile (Fig. 1 and Table S2). It is thought that *Streptomyces* achieves the productivity of a wide variety of secondary metabolites by acquiring foreign biosynthetic enzyme genes through horizontal gene transfer^15^. In GI-I, we found a thiopeptide-lantipeptide biosynthesis pathway, which has the 38% similarity with the cyclothiazomycin biosynthesis pathway (Table 1). Although *S. lydicus* strain 103 and A02 belong to the same species, *S. lydicus* A02 has a larger chromosome (9,300,149 bp) than strain 103 (8,201,357 bp). Besides, the genome size of *S. lydicus* 103 is smaller than two other neighbor species, *Streptomyces bingchenggensis* BCW-1 and *Streptomyces albus* J1074 (Fig. S2). By the distribution of COG classification, we can see that in *S. lydicus* 103 genome, the number of genes related to transcription (K), amino acid transport and metabolism (E), carbohydrate transport and metabolism (G) and the signal transduction mechanisms (T) are more than the other function related genes (Table S3).

Bacterial toxin-antitoxin (TA) system has been identified in *Streptomyces* species, such as 22 putative TA loci in *Streptomyces coelicolor* A3, 27 in *Streptomyces avermitilis* MA-4680 and 14 in *Streptomyces griseus* NBRC 13350. Twenty-eight putative type II TA locus have been predicted in *S. lydicus* 103 genome by TADB, including DUF397, Xre, COG3832 and PIN families (Table S4). Besides, we found the *relBE* locus in *S. lydicus* 103, which rarely existed in *Streptomyces* species. It was reported that over-expression of *S. cattleya* toxin RelE2sca was lethal in *E. coli* and *S. lividans*^16^. The regulatory mechanism of TA loci in *S. lydicus* 103 may be necessary to the environmental stress responses and complex secondary metabolisms.

### The related metabolism of streptolydigin synthesis in *S. lydicus* 103

Primary metabolism significantly influences secondary metabolism and serves as building precursors for antibiotic biosynthesis, including acetyl-CoA, glucose-6-phosphate, glyceraldehyde-3-phosphate, and oxaloacetate^17^. With the aid of KEGG analysis, metabolic network was obtained, including the central carbon metabolism, nitrogen, amino acids and fatty acids metabolism. Among all the KEGG pathways, carbohydrate and amino acid metabolism accounted for the largest proportion.

**Figure 3:**
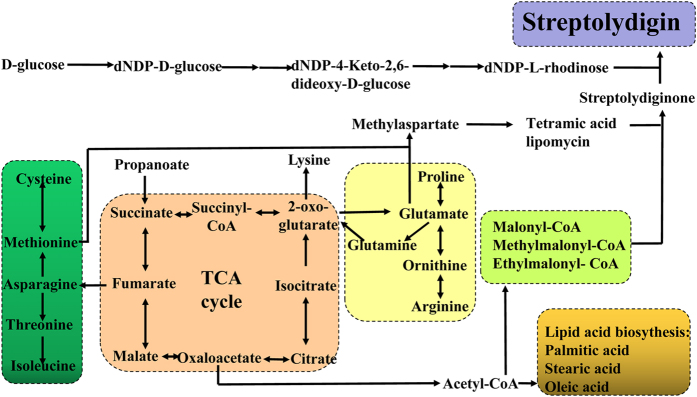
The related metabolism of streptolydigin synthesis in *S. lydicus* 103.

In the central carbon metabolism, *S. lydicus* 103 has the complete glycolysis, citrate cycle and pentose phosphate pathway. Acyl-CoA is the important precursor of acetyl-CoA, malonyl-CoA, methylmalonyl-CoA, and ethylmalonyl-CoA (Fig. 3). Cutting phosphofructokinase would transfer carbon metabolic flux of glycolytic pathway to the pentose phosphate pathway, and acetyl-CoA could be significant accumulated and further converted to antibiotics and pyruvate^18^. In the carbohydrate metabolism, *S. lydicus* 103 harbors the complete pathway that transfers xylitol to D-ribulose-5P, involving pentose phosphate pathway, and contains endoglucanase and beta-glucosidase, which transfer cellulose to glucose. Furthermore, *S. lydicus* 103 contains the PTS system and sugar-specific component, thus utilizing the extracellular trehalose and maltose. In the propanoate metabolism, *S. lydicus* 103 harbors the complete pathway that transfers propanoate to succinate, involving pyruvate metabolism. In the nitrogen metabolism, we found two cycle pathways to transfer CO_2_ and L-glutamate to ammonia, respectively.

In the fatty acids biosynthesis, *S. lydicus* 103 lacks the 3-hydroxyacyl-[acyl-carrier-protein] dehydratase, which is responsible for the dehydration step of the dissociated (type II) fatty-acid biosynthesis system^19^. Moderate control of lipids biosynthesis may distribute more coenzyme A to the streptolydigin biosynthesis. In the fatty acids degradation, *S. lydicus* 103 lacks the O-palmitoyltransferase, which is responsible for the hexadecanoyl-CoA degradation. Besides, *S. lydicus* 103 contains the *tesB* gene that encodes acyl-CoA thioesterase II [EC:3.1.2.-], taking part in biosynthesis of unsaturated fatty acids, e.g. palmitic acid, stearic acid and oleic acid.

Among the amino acids, glutamic acid was the most favorable as the nitrogen source to form streptolydigin^20^. In the glutamine and glutamate metabolism, *S. lydicus* 103 contains the complete pathway to transfer glutamine or glutamate to 2-oxoglutarate, supplying the citrate cycle (Fig. 3). In the cysteine and methionine metabolism, *S. lydicus* 103 harbors the complete pathways to transformation among the L-cysteine, pyruvate, L-homocysteine and L-methionine. L-methionine was not the direct precursor for streptolydigin biosynthesis, but it provided N-methyl of streptolydigin through S-adenosylmethionine, which was catalyzed by S-adanosylmethionine synthase. In the lysine degradation, *S. lydicus* 103 lacks lots of related genes, thus restricting the supplement of acetyl-CoA. In the valine, leucine and isoleucine degradation, *S. lydicus* 103 lacks the 2-oxoisovalerate dehydrogenase E1 component alpha subunit [EC:1.2.4.4] and 2-oxoisovalerate dehydrogenase E2 component (dihydrolipoyl transacylase) [EC:2.3.1.168], thus influencing the biosynthesis of branched chain fatty acid and terpenoid backbone. *S. lydicus* 103 harbors the complete pathways of lysine, valine, leucine, phenylalanine, tyrosine and isoleucine biosynthesis. In addition to the acyl-CoA, L-valine contributes to the biosynthesis for methylmalonyl-CoA and ethylmalonyl-CoA, and L-methionine contributes to the biosynthesis for chloroethylmalonyl-CoA. Proteomics and metabolomics analyses showed that the pitching ratio influenced the activity of glutamate and proline pathways (both precursors of streptolydigin), and exogenously addition can increase the yield of streptolydigin production^21^. We found that *S. lydicus* 103 harbors the complete pathways to transform among the arginine, ornithine, glutamate and proline.

Streptolydigin was a polyketide compound synthesized by type I polyketide pathway, which shares same or similar precursors with the type II polyketide pathways^22^. Complete biosynthetic pathway of streptolydigin has been identified, so that the combined and metabolic processes could be further interpreted^23^. *S. lydicus* 103 harbors 67 ORFs covering a region of 111.2 kb, which are putatively assigned as streptolydigin biosynthesis genes encoding amino-acid permease, isocitrate dehydrogenase, lysophospholipase, erythronolide synthase, phenolphthiocerol synthesis polyketide synthase, cadicidin biosynthesis thioesterase, squalene cyclase, cytochrome P450, methylmalonyl-CoA mutase, glucose-1-phosphate thymidylyltransferase, lipopolysaccharide, biosynthesis protein and electron transfer flavoprotein etc.

### The analysis of secondary metabolite pathways in *S. lydicus* 103

*S. lydicus* can produce a lot of important secondary metabolites, and a total of 27 gene clusters were predicted to be involved in secondary metabolism by antiSMASH. They are mainly focused on polyketide (PKS), nonribosomal peptide (NRPs) and terpene, and most of them have the really low similarity with the known clusters (Table 1).

As the typical PKS I, the biosynthetic pathway of erythromycin has been illuminated, including 6-deoxyerythronolide B (6-dEB) biosynthesis and glycosylation modification^24^. The 6-dEB was condensed by a molecule propionyl CoA and 6 molecules methyl malonyl CoA. The PKS gene cluster of erythromycin contains *eryA* I, *eryA* II and *eryA* III and encodes acyl wansferase, acyl carrier protein, ketosynthase, ketoreductase, dehydratase and enoyl reductase. The improvements of the erythromycin yield by metabolic engineering has been reported^25^. The product of 6-dEB and erythromycin A was reported in titers of 10 mg·L^−1^ 26. As the typical NRPs, mannopeptimycin was first found in industrial bacterium *Streptomyces hygroscopicus*^27^. Mannopeptimycin comprises two distinct stereoisomers of amino acids, thus conforming glycosylated cyclic hexapeptide. Besides, with the different R groups, it can form diverse secondary metabolites. We identified a biosynthetic cluster showing 81% similarity with known mannopeptimycin biosynthetic cluster (BGC0000388_c1), which consists of polyprenyl mannose synthase MppG, polyprenyl phospho-mannosyltransferase MppHI, mannopeptimycin peptide synthetase MppAB, alpha/beta hydrolase MppK, ABC transporter MppL, isovaleryltransferase MppMN, PLP-dependent aminotransferase MppQ, putative transcriptional regulator MppS, hypothetical protein MppT, two component response regulator MppU, two component sensor kinase MppV, hypothetical lipoprotein MppW, ABC transporter MppX, conserved hypothetical protein MppYZ in *S. lydicus* 103.

Besides, *S. lydicus* 103 harbors the ectione biosynthetic pathway that shows 47% similarity with *Streptomyces albulus* PD-1. As one kind of compatible solute, ectoine can be used for protecting enzymes, membranes and whole cells against stresses^28^. The formation of hydroxyectoine in the ectoine producer *Halomonas elongatawas* was improved by the heterologous expression of the ectoine hydroxylase gene from *Streptomyces chrysomallus*^29^. We identify two ectoine dioxygenases (EctD), L-ectoine synthase (EctC), diaminobutyrate-pyruvate aminotransferase (EctB) and L-2,4-diaminobutyric acid acetyltransferase (EctA) in *S. lydicus* 103, which shows 75% similarity with known ectione biosynthetic cluster (BGC0000853_c1). As the family of siderophores, desferrioxamines can form strong hexadentate complexes with ferric iron. Desferrioxamine B has been used for the treatment of iron overload in human^30^. *S. lydicus* 103 harbors the desferrioxamine B biosynthetic pathway that shows 80% similarity with known desferrioxamine B biosynthetic cluster (BGC0000941_c1). Previous research has unambiguously identified desferrioxamine E as the major desferrioxamine siderophore produced by *S. coelicolor* M145 and has identified a cluster of four genes (*des*A-D) that directs desferrioxamine biosynthesis in this model actinomycete^31^. We also identify tetratricopeptide (TPR) protein, DesD-A, HTH domain of SpoOJ/ParA/ParB/repB family, 4-nitrophenylphosphatase, desferrioxamine E transporter and ABC-type Fe^3+^-siderophore transport system in *S. lydicus* 103.

## Discussion

Although streptolydigin produced by *S. lydicus* has the activities mentioned above, the yield from the original strain is not very high yet. To achieve higher antibiotic streptolydigin productivity through metabolic regulation, propionate was fed during the fermentation of *S. lydicus*^32^. The streptolydigin yield, and the carbon fluxes of pentose phosphate pathway and the anaplerotic reaction were significantly increased after propionate feeding. However, it is very difficult to sharply improve the antibiotic production only by the traditional fermentation optimization and mutagenesis treatment. So it is urgent for us to make clear the metabolic network for antibiotic biosynthesis pathways to further improve the production. For example, the cluster *slgE1*-*slgE2*-*slgE3* is involved in 3-methylaspartate (the precursor of the tetramic acid) supply. SlgE3, a ferredoxin-dependent glutamate synthase, is responsible for the biosynthesis of glutamate from glutamine and 2-oxoglutarate. The expression of *slgE3* is increased up to 9-fold at the onset of streptolydigin biosynthesis^33^. The asparaginyl-tRNA synthetase-like SlgZ and methyltransferase SlgM enzymes are involved in the biosynthesis of the tetramic acid in *S. lydicus*. Over-expression of *slgZ* and *slgM* in *S. lydicus* led to a considerable increase in streptolydigin production^34^. *SlnM* gene overexpression with different promoters can improve the natamycin production in *S. lydicus* A02^35^. The biosynthetic genes or regulatory elements of a metabolite must be characterized prior to metabolic engineering^36^. Furthermore, modifications to the structures of secondary metabolites can often change the biological activity of the compound^37^. In this study, we presented the complete genome sequence of *S. lydicus* 103 and identified the pathways related to streptomyces biosynthesis from primary metabolism to secondary metabolite, which would provide more accurate analysis of the metabolic network and a more rational adjustment of metabolic regulation^38^.

Genomics-based bottom-up approaches have been developed to unveil biosynthetic pathways of new natural products that were undetected under standard fermentation conditions^39^. Despite being tapped as antibiotic sources for decades, *Streptomyces* spp. could produce up to 100,000 antimicrobial metabolites, while only a small proportion have been identified^40^. As an example, a terpene synthase from *S. avermitilis* was expressed in *E. coli*, resulting in the synthesis of the novel tricyclic sesquiterpene, avermitilol^41^. Chu *et al*.^42^ used primary sequence from the human microbiome, and thus bioinformatically predicted and chemical synthesized a new antibiotic. Luo *et al*.^43^ applied a plug-and-play synthetic biology strategy to activate a cryptic polycyclic tetramate macrolactams (PTMs) biosynthetic gene cluster from *S. griseus* and discovered three new PTMs. Besides, transcriptome and metabolome can identify the potential biosynthetic genes by correlating the expression of the secondary metabolite related gene^44^. In *S. lydicus* 103, we found many new gene clusters that have really low similarity with known clusters (Table 1). Thus, further studies are desirable for optimization, isolation and identification of the new bio-active molecule. The availability of the genome sequence of *S. lydicus* 103 provides a framework for biotechnological analysis and characterization of new natural products.

## Methods

### Bacterial culture and genome sequence

*S. lydicus* 103, an actinomycete, was isolated from soil. One loop of cells was incubated in a 250 mL flask containing 50 mL seed medium for 48 hours at 28 °C with shaking at 220 r·min^−1^. The seed medium contained (g·L^−1^): glucose 5, starch 30, yeast extract 2, peptone 4, K_2_HPO_4_ 1.5, NaCl 0.5, and MgSO_4_.7H_2_O 0.5. Isolation of genomic DNA was carried out using SDS method. Total DNA obtained was subjected to quality control by agarose gel electrophoresis and quantified by Qubit. The genome was sequenced by Single Molecule, Real-Time (SMRT) technology. Sequencing was performed at the Beijing Novogene Bioinformatics Technology Co., Ltd. SMRT Analysis 2.3.0 was used to filter low quality reads and the filtered reads were assembled to the chromosome without gaps. The circular skeleton of chromosome was identified by the long fragment across the head and tail.

### Genome annotation and bioinformatics analysis

Transfer RNA (tRNA) genes, Ribosome RNA (rRNA) genes, small RNA (sRNA) genes were predicted with tRNAscan-SE^45^, rRNAmmer^46^ and Rfam database^47^, respectively. Gene prediction was performed with the integrated model by NCBI prokaryotic annotation pipeline^48^, and gene functional prediction was performed by Blast^49^ against the databases, KEGG^50^ (Kyoto Encyclopedia of Genes and Genomes), COG^51^ (Clusters of Orthologous Groups), Swiss-Prot^52^, and GO^53^ (Gene Ontology). The origin of replication (*oriC*) and putative DnaA boxes were identified using Ori-Finder^54^. GC-Profile was used to compute the GC content variation in genome sequence and predict the genomic islands^55^. CGView Server^56^, a comparative genomics tool for circular genomes, was used to obtain a circular graphical representation of chromosome. A whole genome-based, alignment-free composition vector (CV) method was performed for phylogenetic analysis^57^ and the phylogenetic tree was generated using the MEGA program^58^. The toxin-antitoxin (TA) systems were predicted by TADB^59^. Secondary metabolite gene clusters were predicted by antiSMASH^60^. BioVenn, a web application for the comparison and visualization of biological lists, was used for Venn diagrams drawing^61^.

### GenBank accession number

The sequence of the *S. lydicus* 103 genome has been deposited at DDBJ/EMBL/GenBank under the GenBank accession number CP017157.

## Additional Information

**How to cite this article:** Jia, N. *et al*. Complete genome sequencing and antibiotics biosynthesis pathways analysis of *Streptomyces lydicus* 103. *Sci. Rep.* **7**, 44786; doi: 10.1038/srep44786 (2017).

**Publisher's note:** Springer Nature remains neutral with regard to jurisdictional claims in published maps and institutional affiliations.

## Supplementary Material

Supplementary Information

## Figures and Tables

**Table 1:** Putative gene clusters coding for secondary metabolites in *S. lydicus* 103.

Type	From (bp)	To (bp)	Most similar known cluster	Similarity*
Lantipeptide	140342	176012	Chlorizidine A biosynthesis	11%
	3656781	3679393	SapB biosynthesis	100%
Lassopeptide	1043276	1065896	—	—
NRPs	4508815	4560693	A-500359s biosynthesis	10%
	7791219	7850756	Mannopeptimycin biosynthesis	81%
Ectoine	2288536	2299192	Ectoine biosynthesis	75%
Siderophore	2380919	2392976	Desferrioxamine B biosynthesis	80%
	6256663	6271630	—	—
Bacteriocin	3366951	3377184	—	—
	4574162	4586126	—	—
	5991578	6003917	—	—
Terpene	4485349	4511671	Isorenieratene biosynthesis	100%
	5409699	5436377	Hopene biosynthesis	69%
	5579311	5600591	Kanamycin biosynthesis	46%
	7923608	7945845	Salinomycin_biosynthesis	4%
Butyrolactone	4723717	4734682	Hygrocin biosynthesis	6%
	6059828	6070904	—	—
Other	5055818	5097455	A-503083 biosynthesis	7%
T1pks	632282	678623	—	—
T2pks	3269415	3311930	Spore pigment biosynthesis	83%
T1pks-NRPs	679064	735066	Erythromycin biosynthesis	8%
T1pks-Terpene-NRPs	2855210	2966492	Streptolydigin biosynthesis	97%
NRPs-T1pks	3761274	3830863	SW-163 biosynthesis	10%
NRPs-T3pks	6090695	6154188	Arylomycin biosynthesis	55%
NRPs-Melanin	4933499	4994082	WS9326 biosynthesis	10%
Thiopeptide-Lantipeptide	3985339	4037579	Cyclothiazomycin biosynthesis	38%
Lassopeptide-NRPs-Nucleoside	5150871	5208226	Toyocamycin biosynthesis	30%

Secondary metabolite types detected by antiSMASH: **T1pks** Type I PKS cluster; **T2pks** Type II PKS cluster; **T3pks** Type III PKS cluster; **NRPs** Nonribosomal peptide synthetase cluster; **Bacteriocin** Bacteriocin or other unspecified ribosomally synthesis and post-translationally modified peptide product (RiPP) cluster; **Lassopeptide** Lasso peptide cluster; **Other** cluster containing a secondary metabolite- related protein that does not fit into any other category.

^*^The “similarity” means the percentage of the homologous genes in the query cluster that are present in the hit cluster. According to the defination by the antiSMASH, the homologous genes were selected by BLAST e-value < 1E-05, 30% minimal sequence identity, shortest BLAST alignment covers over >25% of the sequence.
