# Supplementary material for: Complete genome sequencing and antibiotics biosynthesis pathways analysis of Streptomyces lydicus 103
Source: Sci Rep. 2017 Mar 20;7:44786. doi: 10.1038/srep44786 (PMC5357945; doi:10.1038/srep44786)
Supplement: Supplementary Information [file srep44786-s1.doc]

**Supplementary information**

**Complete genome sequencing and antibiotics biosynthesis pathways analysis of *Streptomyces lydicus* 103**

Nan Jia^1, 2^, Ming-Zhu Ding^1, 2*^, Hao Luo^1, 2, 3^, Feng Gao^1, 2, 3*^, Ying-Jin Yuan^1, 2^

1. Key Laboratory of Systems Bioengineering (Ministry of Education), School of Chemical Engineering and Technology, Tianjin University, Tianjin, 300072, PR China
2. SynBio Research Platform, Collaborative Innovation Centre of Chemical Science and Engineering (Tianjin), School of Chemical Engineering and Technology, Tianjin University, Tianjin, 300072, PR China.
3. Department of Physics, Tianjin University, Tianjin, 300072, PR China

* Corresponding authors: Ming-Zhu Ding, Feng Gao

Email: [mzding@tju.edu.cn](mailto:yjyuan@tju.edu.cn)[,](mailto:fgao@tju.edu.cn,) [fgao@tju.edu.cn](mailto:fgao@tju.edu.cn)

**
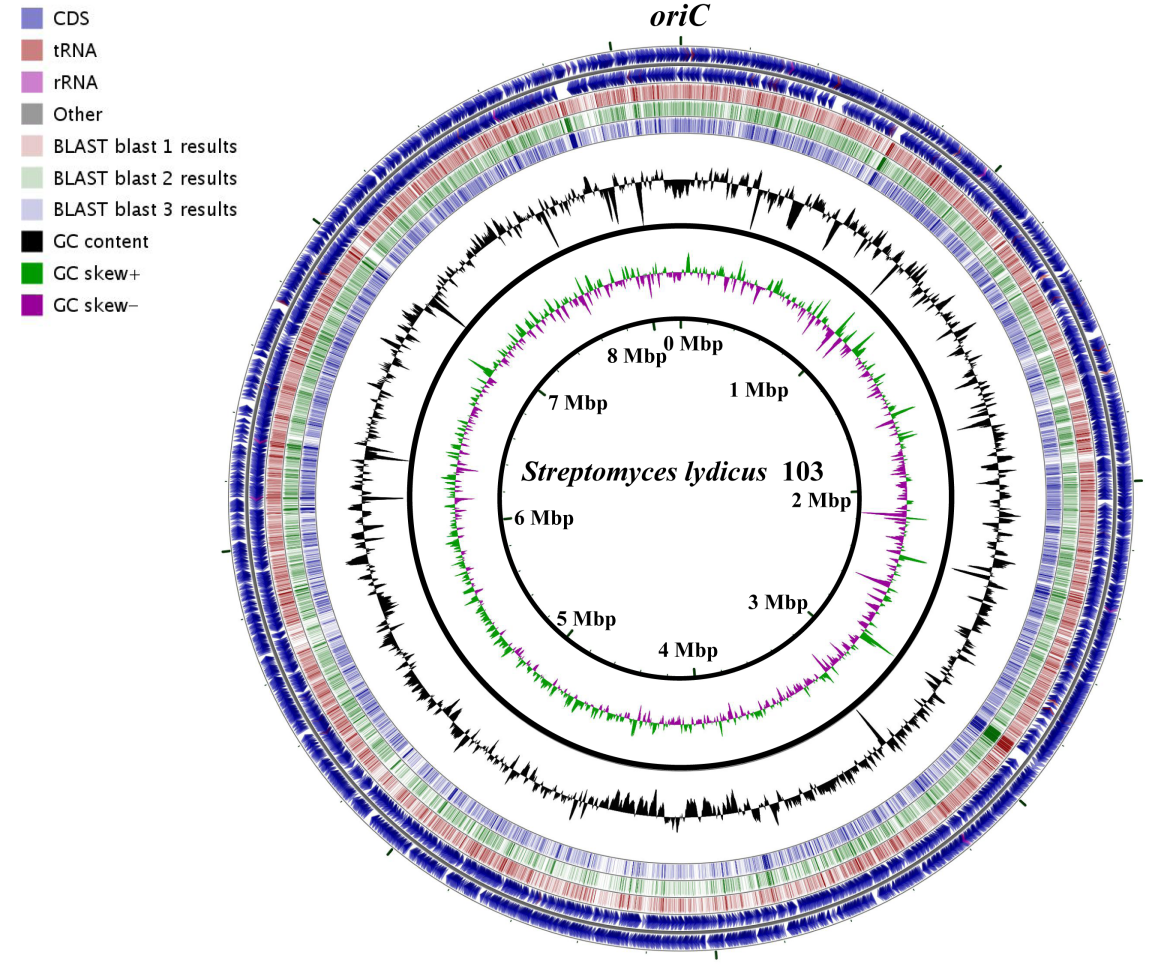
**

**Fig. S1. The genome structure of *S. lydicus* 103.** Circles from the outside in show: the positions of protein-coding genes (blue), tRNA genes (red) and rRNA genes (pink) on the positive (circle 1), and negative (circle 2), strands respectively. Circles 3-5 show the positions of BLAST hits detected through blastx comparisons of the chromosome sequence of *S. lydicus* 103 against those of *S. lydicus* A02 (circle 3), *S. bingchenggensis* BCW-1 (circle 4) and *S. albus* J1074 (circle 5). The height of the shading in the BLAST results rings is proportional to the percent identity of the hit. Circles 6 and 7 show plots of GC content and GC skew plotted as the deviation from the average for the entire sequence.


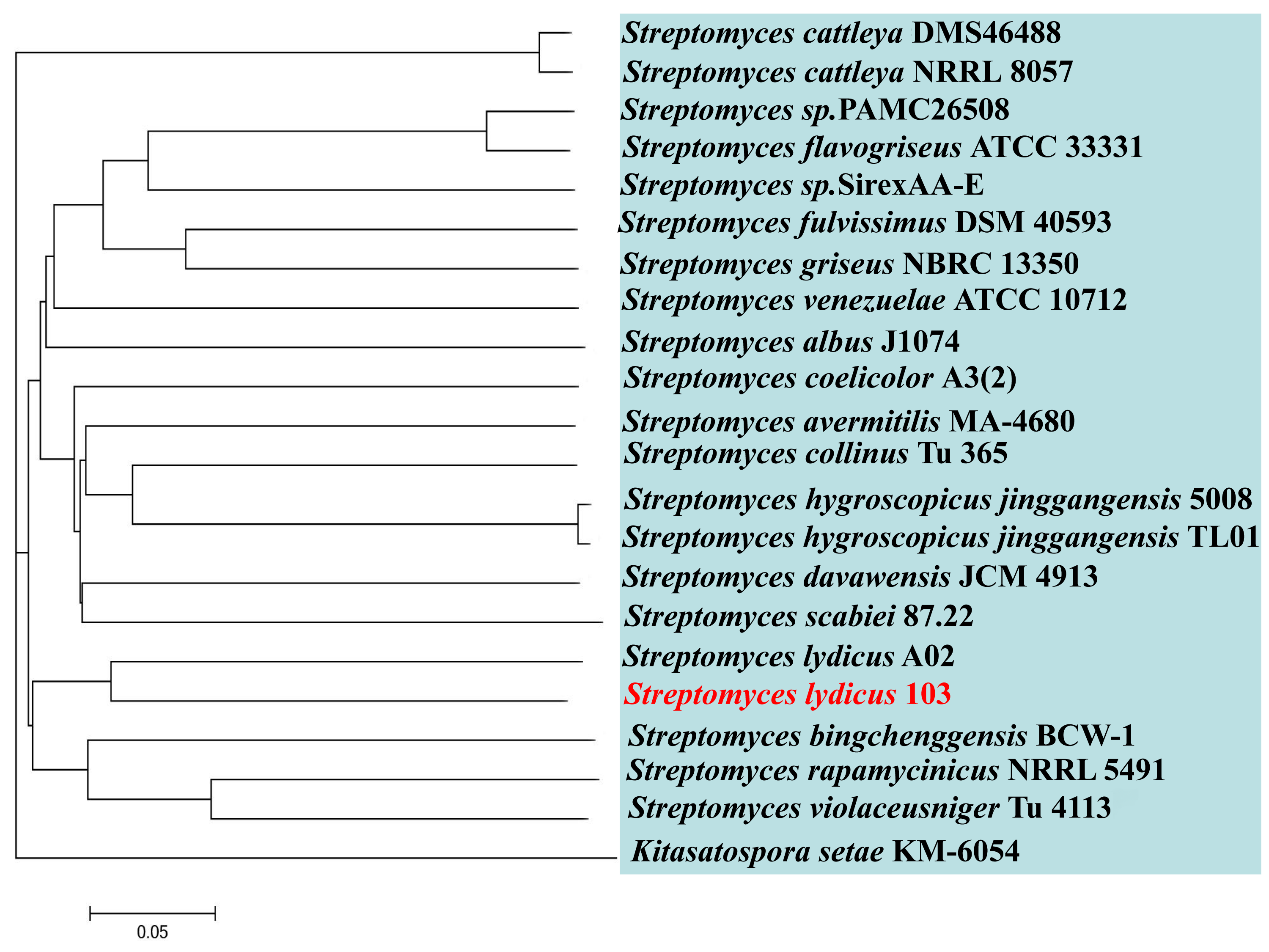


**Fig. S2. Phylogenetic analysis of *S. lydicus* 103 with other *Streptomyces* species.** The phylogenetic tree of *S. lydicus* 103 was constructed using CVTree with parameters: K=6, Type=aa, and the neighbour-joining tree was constructed using the MEGA5 program. Note that *Kitasatospora setae* KM-6054 was included as outgroup.

**Table S1. general features of the chromosome of *S. lydicus* 103.**

| **Feature** |  |
| --- | --- |
| **Length of sequence (bp)** | 8,201,357 |
| **G+C content (%)** | 72.22 |
| **Gene** | |
| **Protein-coding gene number** | 6,872 |
| **Total length of genes (bp)** | 7,016,157 |
| **Average gene length (bp)** | 965 |
| **Gene length/Genome (%)** | 85.32 |
| **RNA** | |
| **5s rRNA** | 7 |
| **16s rRNA** | 7 |
| **23s rRNA** | 7 |
| **tRNA** | 67 |
| **sRNA** | 9 |

**Table S2. The results of GC-Profile segmentation in *S. lydicus* 103.**

| Start | End | Length (bp) | GC content (%) | No. of core genes | No. of unique genes | Total genes |
| --- | --- | --- | --- | --- | --- | --- |
| 1 | 35856 | 35856 | 69.64 | 6 | 0 | 26 |
| 35857 | 297152 | 261296 | 72.25 | 19 | 2 | 225 |
| 297153 | 324303 | 27151 | 74.99 | 1 | 1 | 18 |
| 324304 | 326354 | 2051 | 69.28 | 0 | 0 | 1 |
| 326355 | 332359 | 6005 | 57.2 | 0 | 0 | 1 |
| 332360 | 433099 | 100740 | 70.73 | 18 | 1 | 92 |
| 433100 | 476885 | 43786 | 73.31 | 11 | 0 | 38 |
| 476886 | 518713 | 41828 | 66.67 | 41 | 0 | 51 |
| 518714 | 600237 | 81524 | 73.91 | 10 | 0 | 70 |
| 600238 | 669344 | 69107 | 72.31 | 1 | 0 | 47 |
| 669345 | 687576 | 18232 | 65.77 | 0 | 1 | 15 |
| 687577 | 2851129 | 2163553 | 72.55 | 237 | 17 | 1782 |
| 2851130 | 2857696 | 6567 | 65.8 | 0 | 0 | 6 |
| 2857697 | 2906240 | 48544 | 70.94 | 1 | 0 | 19 |
| 2906241 | 3812416 | 906176 | 73.2 | 11 | 1 | 792 |
| **3812417** | **4085811** | **273395** | **70.28** | **1** | **4** | **220*** |
| 4085812 | 4171989 | 86178 | 73.3 | 0 | 1 | 74 |
| **4171990** | **4395111** | **223122** | **69.55** | **2** | **7** | **176*** |
| 4395112 | 4485856 | 90745 | 71.16 | 1 | 1 | 83 |
| 4485857 | 5040326 | 554470 | 72.43 | 8 | 7 | 496 |
| 5040327 | 5136149 | 95823 | 73.93 | 0 | 0 | 85 |
| 5136150 | 5239518 | 103369 | 70.86 | 1 | 0 | 82 |
| 5239519 | 5642931 | 403413 | 73.31 | 13 | 2 | 338 |
| 5642932 | 5753144 | 110213 | 70.25 | 1 | 1 | 91 |
| 5753145 | 6016367 | 263223 | 73.05 | 11 | 1 | 216 |
| 6016368 | 6041154 | 24787 | 68.86 | 5 | 0 | 15 |
| 6041155 | 6863245 | 822091 | 71.87 | 118 | 2 | 668 |
| 6863246 | 6980211 | 116966 | 74 | 8 | 0 | 102 |
| 6980212 | 6985299 | 5088 | 65.29 | 0 | 0 | 4 |
| 6985300 | 7019013 | 33714 | 71.21 | 1 | 0 | 25 |
| 7019014 | 7038012 | 18999 | 64.2 | 1 | 1 | 17 |
| 7038013 | 7079316 | 41304 | 70.94 | 7 | 0 | 37 |
| 7079317 | 7291280 | 211964 | 72.57 | 21 | 1 | 187 |
| 7291281 | 7449106 | 157826 | 74.13 | 16 | 0 | 125 |
| 7449107 | 7802045 | 352939 | 71.86 | 49 | 5 | 314 |
| 7802046 | 7867797 | 65752 | 73.78 | 3 | 0 | 41 |
| 7867798 | 7902752 | 34955 | 69.25 | 0 | 0 | 27 |
| 7902753 | 7923621 | 20869 | 65.73 | 0 | 1 | 13 |
| 7923622 | 7942052 | 18431 | 75.01 | 0 | 0 | 14 |
| 7942053 | 8017832 | 75780 | 72.19 | 4 | 0 | 82 |
| 8017833 | 8026357 | 8525 | 64.59 | 0 | 0 | 6 |
| 8026358 | 8030364 | 4007 | 57.05 | 0 | 0 | 2 |
| 8030365 | 8041034 | 10670 | 70.32 | 0 | 0 | 8 |
| 8041035 | 8131305 | 90271 | 72.94 | 2 | 2 | 75 |
| 8131306 | 8201357 | 70052 | 69.5 | 12 | 0 | 66 |

* The predicted genomic islands have been highlighted in bold.

**Table S3. The COG distribution of all identified genes in *S. lydicus* 103.**

| Functional class | Class description | Gene number |
| --- | --- | --- |
| A | RNA processing and modification | 1 |
| B | Chromatin structure and dynamics | 2 |
| C | Energy production and conversion | 329 |
| D | Cell cycle control, cell division, chromosome partitioning | 63 |
| E | Amino acid transport and metabolism | 520 |
| F | Nucleotide transport and metabolism | 141 |
| G | Carbohydrate transport and metabolism | 466 |
| H | Coenzyme transport and metabolism | 341 |
| I | Lipid transport and metabolism | 354 |
| J | Translation, ribosomal structure and biogenesis | 279 |
| K | Transcription | 735 |
| L | Replication, recombination and repair | 147 |
| M | Cell wall/membrane/envelope biogenesis | 263 |
| N | Cell motility | 20 |
| O | Posttranslational modification, protein turnover, chaperones | 207 |
| P | Inorganic ion transport and metabolism | 275 |
| Q | Secondary metabolites biosynthesis, transport and catabolism | 278 |
| R | General function prediction only | 723 |
| S | Function unknown | 275 |
| T | Signal transduction mechanisms | 427 |
| U | Intracellular trafficking, secretion, and vesicular transport | 43 |
| V | Defense mechanisms | 194 |
| W | Extracellular structures | 12 |
| X | Mobilome: prophages, transposons | 26 |

**Table S4. The predicted TA II system in *S. lydicus* 103.**

| No | ID | T/A | location | Length  (a. a.) | strand | family | domain | Score TA |
| --- | --- | --- | --- | --- | --- | --- | --- | --- |
| 1 | 641929726  641929727 | T  A | 79341..80201  80315..80998 | 286  227 | +  + |  | COG2153  pfam00392 | 17.72 |
| 2 | 641930004  641930003 | T  A | 399292..399507  398460..399311 | 71  283 | +  + | DUF397-like  Xre-like | pfam04149  Pfam13560 | 47.89 |
| 3 | 641930223  641930224 | T  A | 636422..636631  636830..637696 | 69  288 | -  - | DUF397-like  Xre-like | pfam04149  Cd00093 | 67.59 |
| 4 | 641930283  641930282 | T  A | 719809..721329  719036..719698 | 506  220 | +  + |  | pfam12568  pfam00440 | 6.8 |
| 5 | 641930415  641930416 | T  A | 872898..873167  873164..873436 | 89  90 | -  - | relE-like  relB | COG2026  COG4118 | 40.03 |
| 6 | 641930755  641930756 | T  A | 1280208..1281344  1281370..1282434 | 378  354 | -  - |  | COG1246  cd01392 | 11.55 |
| 7 | 641930939  641930940 | T  A | 1518086..1518379  1518361..1519194 | 97  277 | -  - | DUF397-like  Xre-like | pfam04149  pfam13560 | 62.42 |
| 8 | 641930978  641930977 | T  A | 1569747..1570250  1569331..1569750 | 167  139 | -  - | COG3832-like  ArsR-like | cd07814  cd00090 | 43.99 |
| 9 | 641930989  641930990 | T  A | 1582819..1583106  1583103..1583933 | 95  276 | -  - | DUF397-like  Xre-like | pfam04149  pfam13560 | 40.79 |
| 10 | 641931242  641931243 | T  A | 1843689..1845308  1845305..1845967 | 539  220 | +  + |  | pfam12568  pfam00440 | 9.1 |
| 11 | 641931264  641931265 | T  A | 1871554..1871745  1871742..1872584 | 63  280 | -  - | DUF397-like  Xre-like | pfam04149  pfam13560 | 58.06 |
| 12 | 641931680  641931679 | T  A | 2352056..2352256  2351214..2352059 | 66  281 | +  + | DUF397-like  Xre-like | pfam04149  pfam13560 | 41.02 |
| 13 | 641931996  641931995 | T  A | 2705179..2706777  2704631..2705182 | 532  183 | +  + |  | pfam12568  TIGR02684 | 5.43 |
| 14 | 641932227  641932228 | T  A | 2995658..2996131  2996264..2997109 | 157  281 | -  - |  | pfam10604  COG5625 | 15.96 |
| 15 | 641932601  641932602 | T  A | 3404416..3404652  3404820..3405731 | 78  303 | +  + |  | pfam04149  pfam01381 | 12.67 |
| 16 | 641933871  641933872 | T  A | 4795125..4795496  4795493..4795768 | 123  91 | -  - | Doc  RHH-like |  | 72.76 |
| 17 | 641933959  641933958 | T  A | 4882653..4883501  4882339..4882656 | 282  105 | +  + | COG3832-like  ArsR-like | pfam10604  cd00090 | 51.21 |
| 18 | 641934421  641934422 | T  A | 5410788..5411066  5411074..5411940 | 92  288 | -  - | DUF397-like  Xre-like | pfam04149  pfam13560 | 65.17 |
| 19 | 641934447  641934446 | T  A | 5439093..5440064  5438393..5439037 | 323  214 | -  - | PIN-like  Xre-like |  | 18.09 |
| 20 | 641934609  641934608 | T  A | 5625537..5627027  5625109..5625540 | 496  143 | +  + |  | pfam12568  pfam01047 | 4.05 |
| 21 | 641935399  641935400 | T  A | 6576248..6577660  6577862..6578302 | 470  146 | -  - |  | pfam12568  pfam01047 | 3.43 |
| 22 | 641935407  641935406 | T  A | 6583756..6584010  6582938..6583759 | 84  273 | +  + | DUF397-like  Xre-like | pfam04149  pfam13560 | 49.44 |
| 23 | 641936137  641936136 | T  A | 7415048..7415263  7414001..7414840 | 71  279 | +  + | DUF397-like  Xre-like | pfam04149  cd00093 | 49.52 |
| 24 | 641936339  641936338 | T  A | 7629224..7629436  7628400..7629242 | 70  280 | +  + | DUF397-like  Xre-like | pfam04149  pfam13560 | 58.79 |
| 25 | 641936367  641936368 | T  A | 7664279..7664797  7664807..7665307 | 172  166 | -  - |  | COG1246  COG0735 | 14.64 |
| 26 | 641936610  641936611 | T  A | 7951628..7951855  7951848..7952642 | 75  264 | -  - | DUF397-like  Xre-like | pfam04149  cd00093 | 68.99 |
| 27 | 641936672  641936671 | T  A | 8001449..8003041  8000475..8001263 | 530  262 | -  - |  | pfam12568  pfam00440 | 5.46 |
| 28 | 641936813  641936812 | T  A | 8157329..8157781  8156700..8157233 | 150  177 | +  + |  | PLN02706  pfam01047 | 9.93 |

T/A: This shows the type of the protein. T for Toxin and A for Antitoxin
